# The impact of preoperative language mapping by repetitive navigated transcranial magnetic stimulation on the clinical course of brain tumor patients

**DOI:** 10.1186/s12885-015-1299-5

**Published:** 2015-04-11

**Authors:** Nico Sollmann, Sebastian Ille, Theresa Hauck, Stefanie Maurer, Chiara Negwer, Claus Zimmer, Florian Ringel, Bernhard Meyer, Sandro M Krieg

**Affiliations:** 1 Department of Neurosurgery, Klinikum rechts der Isar, Technische Universität München, Ismaninger Str. 22, 81675 Munich, Germany; 2 TUM-Neuroimaging Center, Klinikum rechts der Isar, Technische Universität München, Ismaninger Str. 22, 81675 Munich, Germany; 3 Section of Neuroradiology, Department of Radiology, Klinikum rechts der Isar, Technische Universität München, Ismaninger Str. 22, 81675 Munich, Germany

**Keywords:** Awake surgery, Direct cortical stimulation, Matched pair, Preoperative mapping, Transcranial magnetic stimulation

## Abstract

**Background:**

Language mapping by repetitive navigated transcranial magnetic stimulation (rTMS) is used for resection planning in patients suffering from brain lesions within regions known to be involved in language function. Yet we also need data that show whether patients benefit clinically from preoperative rTMS for language mapping.

**Methods:**

We enrolled 25 patients with language eloquently located brain lesions undergoing preoperative rTMS language mapping (GROUP 1, 2011–2013), with the mapping results not being available for the surgeon, and we matched these patients with 25 subjects who also underwent preoperative rTMS (GROUP 2, 2013–2014), but the mapping results were taken into account during tumor resection. Additionally, cortical language maps were generated by analyzing preoperative rTMS and intraoperative direct cortical stimulation (DCS) data.

**Results:**

Mean anterior-posterior (ap) craniotomy extents and overall craniotomy sizes were significantly smaller for the patients in GROUP 2 (Ap: p = 0.0117; overall size: p = 0.0373), and postoperative language deficits were found significantly more frequently for the patients in GROUP 1 (p = 0.0153), although the preoperative language status did not differ between groups (p = 0.7576). Additionally, there was a trend towards fewer unexpected tumor residuals, shorter surgery duration, less peri- or postoperative complications, shorter inpatient stay, and higher postoperative Karnofsky performance status scale (KPS) for the patients in GROUP 2.

**Conclusions:**

The present study provides a first hint that the clinical course of patients suffering from brain tumors might be improved by preoperative rTMS language mapping. However, a significant difference between both groups was only found for craniotomy extents and postoperative deficits, but not for other clinical parameters, which only showed a trend toward better results in GROUP 2. Therefore, multicenter trials with higher sample sizes are needed to further investigate the distinct impact of rTMS language mapping on the clinical course of brain tumor patients.

## Background

Recently, various studies have reported on repetitive navigated transcranial magnetic stimulation (rTMS) being performed prior to brain tumor resection in order to generate individual language maps [1-5]. In that context, it was already shown that rTMS-based cortical language mapping is a safe and tolerable procedure for the patient, which is highly reliable in obtaining maps of language distribution especially when outlining language-negative brain areas as a negative mapping [1]. Basically, the rTMS technique uses the principle of electromagnetic induction: A magnetic coil induces a transient magnetic field penetrating the skull, which triggers the generation of a perpendicularly orientated electrical field [6,7]. The induced electrical field is able to transiently modulate cortical neuronal activation, and, when applied during an object naming task, this neuronal modulation can cause transient, audibly detectable impairment of language [4,8]. In selected cases, this approach was even superior to functional magnetic resonance imaging (fMRI) for detection of language-related cortical areas [2]. Although it is increasingly used for presurgical planning in neurosurgery, data about the impact of rTMS language mapping on the clinical course and outcome parameters are still lacking, and the potential of this comparatively new modality has not yet been under systematical investigation.

For the present study, we assumed that the availability of preoperatively gained rTMS language mapping data could principally have a positive influence on the patients’ clinical course. We therefore compared two patient groups who underwent preoperative rTMS, but only the mapping results of one group were available for the surgeon during tumor resection. In addition, this study provides cortical language maps generated by preoperative rTMS and intraoperative direct cortical stimulation (DCS).

## Methods

### Enrolled patients

Twenty-five consecutive patients suffering from language eloquently located brain lesions within the left hemisphere were enrolled from April 2011 to January 2013 (GROUP 1), and underwent preoperative rTMS language mapping followed by lesion resection in our department. However, the language mapping results were not available for the surgeon during surgery.

This cohort was matched with a group of another 25 patients also suffering from lesions within the perisylvian regions of the left hemisphere (GROUP 2). Subjects in this group underwent surgery between February 2013 and July 2014 in our department by the same surgeons (BM and FR), but—in contrast to GROUP 1—rTMS language mapping results for each patient were available for the surgeon during the operation within the neuronavigation system (BrainLAB Curve, BrainLAB AG, Feldkirchen, Germany).

### Ethical standard

The presented study is in accordance with ethical standards outlined in the Declaration of Helsinki. The study protocol was also approved by the local institutional review board of the Technische Universität München (registration number: 2793/10). All patients gave written informed consent prior to the rTMS investigation.

### Clinical assessment

Each patient initially underwent a detailed examination according to a standardized protocol that included sensory function, coordination, muscle strength, cranial nerve function, and language function. The neurological status was again assessed for each patient directly after surgery and daily from the first postoperative day until discharge, again at 6–8 weeks postoperatively, and during follow-ups every 3–12 months depending on the type of brain lesion. We additionally determined the individual pre- and postoperative Karnofsky performance status (KPS) of each patient. For pre- and postoperative language evaluation, two deficit grades were distinguished:

- None to mild deficit (undisrupted conversational speech and speech comprehension, adequate communication ability to slight amnesic aphasia)
- Medium to severe deficit (impairment of conversational speech and/or speech comprehension, disrupted communication ability)

### Magnetic resonance imaging

Preoperative magnetic resonance imaging (MRI) scans were performed in all patients with a 3 Tesla MR scanner with an 8-channel phased array head coil (Achieva 3 T, Philips Medical Systems, The Netherlands B.V.). Our standard protocol included contrast-enhanced 3D gradient echo sequence, T2 FLAIR, and diffusion tensor imaging (DTI). The contrast-enhanced 3D gradient echo sequence dataset was transferred to the rTMS system for navigation purposes during language mapping sessions (eXimia 3.2 and eXimia 4.3, Nexstim Oy, Helsinki, Finland).

The day after surgery, all patients again underwent MRI on the same scanner in order to evaluate the extent of resection (EOR) and potential surgery-related complications. The protocol included T1 sequences with and without contrast enhancement, T2 FLAIR, and diffusion-weighted imaging (DWI) to identify surgery-related ischemic events.

Furthermore, MRI scans were performed during regular follow-up every 3–12 months depending on the brain lesion type and current oncological treatment, and these scans were reviewed for recurrent tumors.

### Preoperative rTMS language mapping

#### Experimental setup and threshold determination

All rTMS language mapping sessions were performed with the Nexstim eXimia NBS system (Version 3.2 and 4.3) combined with a NexSpeech® module (Nexstim Oy, Helsinki, Finland). Our principal setup follows the reports of previous studies on rTMS language mapping [1-5,8,9].

First, each patient underwent the same procedure to determine the individual resting motor threshold (RMT) by motor mapping of the right abductor pollicis brevis muscle, as described in earlier reports [10,11]. Subsequently, language mapping was carried out with a stimulation intensity related to the individual RMT.

#### Object naming and baseline testing

As a common and frequently used task in neurosurgery, object naming was used for baseline testing as well as rTMS language mapping in the present study [12]. This approach has already been described frequently [1-5,8,9].

For baseline testing, 131 colored photographs of familiar objects, which were provided by the NexSpeech® module, were displayed on a screen in front of the patient at an inter-picture interval of 2.5 s and a display time of 0.7 s without simultaneous stimulation. Every subject was instructed to name all objects as precisely and quickly as possible in his/her mother tongue. Misnamed objects were discarded from the sequence.

After the first baseline testing session, a second one with the stack of remaining images was carried out in an analogue way. Only the remaining objects were then used for language mapping.

#### Mapping procedure and video analysis

The set of objects named correctly according to the baseline testing was displayed time-locked to a train of rTMS pulses. The magnetic coil was moved manually after each image in steps of approximately 10 mm over the left hemisphere and placed tangential to the skull in strict anterior-posterior (ap) field orientation to achieve maximum field induction [13,14]. By causing a virtual functional lesion, rTMS is able to elicit different kinds of naming errors and can thereby identify cortical regions related to language function. All mapping sessions and baseline performances were digitally video recorded [8]. Furthermore, each patient was asked to rate any perceived pain due to rTMS according to the visual analogue scale (VAS). After the mapping sessions, the videos were examined by the same person who had already performed language mapping. Any no response, which is defined as a complete lack of naming response during stimulation, was picked out of the subjects’ videos. Then, all of these naming errors were compared to the corresponding baseline performance, and then the double-checked, no-response errors were counted [8]. These cortical points were then exported from the rTMS system in DICOM standard and imported into the neuronavigation planning software (BrainLAB iPlan® Net Cranial 3.0.1; BrainLAB AG, Feldkirchen, Germany).

### Surgical setup

Generally, the surgical technique and surgeons did not vary between both groups. The principals of the awake surgery approach for intraoperative language mapping by DCS were described in previous publications [1,9,15].

In short, total intravenous anesthesia was used by continuous propofol administration. Furthermore, intraoperative analgesia was guaranteed by continuous administration of remifentanyl. Regarding intraoperative language mapping, DCS as well as subcortical mapping was performed in all patients. The sites of cortical stimulation were placed about 10 mm apart, and DCS (0–10 mA, 50/60 Hz, 4 s duration) was carried out using a bipolar electrode with 1 mm diameter tips separated by a distance of 5 mm. The triggering of object presentation and DCS onset was performed by an audio signal, as this allowed the neurosurgeon to place the electrode on the brain immediately with object presentation.

For patients in GROUP 2, the language-positive sites according to the preoperative rTMS language mapping were visualized as 3D objects by fusion and simple auto segmentation within the neuronavigation data set (BrainLAB iPlan® Net Cranial 3.0.1; BrainLAB AG, Feldkirchen, Germany).

### Data analysis

A Chi-square or Fisher Exact test was performed to test the distribution of several attributes. Furthermore, a t-test (for parametric distribution) was used for testing the differences between both groups. A p-value <0.05 was considered significant. All results are presented as total numbers, percentages, medians, mean values ± standard deviation (SD), or as 95% confidence intervals (CI) (GraphPad Prism 5.0c, La Jolla, CA, USA).

For the visualization of no-response errors elicited by rTMS or DCS, error rates for all cortical regions stimulated were calculated, which were then pooled across subjects and projected into the cortical parcellation system (CPS) [16]. Each of these rates represents the number of individuals with no-response errors in a certain CPS region divided by the number of stimulated patients and is then provided as a percentage. As this approach can lead to a loss of spatial resolution, only the exact language-positive cortical spots, which were directly transferred from the rTMS system to the neuronavigation planning software (BrainLAB iPlan® Net Cranial 3.0.1; BrainLAB AG, Feldkirchen, Germany), were available during surgery of GROUP 2 patients.

## Results

### Characteristics of patients and lesions

Subject-related characteristics including age, gender, lesion histology, lesion diameter, initial KPS scores, and preoperative language function status are provided in Table 1 for both patient groups. In 15 out of 25 patients of each group, the intracranial lesion primarily affected anterior language-related areas (classic Broca’s area and surrounding brain regions: anterior, middle, posterior middle frontal gyrus; orbital, triangular, opercular inferior frontal gyrus; middle and ventral precentral gyrus), whereas the remaining 10 patients of each group were diagnosed with lesions located within posterior language-related brain regions (classic Wernicke’s area and surrounding brain regions: ventral postcentral gyrus; anterior and posterior supramarginal gyrus; angular gyrus; anterior, middle, posterior superior temporal gyrus; anterior, middle, posterior middle temporal gyrus).

**Table 1:** **Patient data**

		GROUP 1	GROUP 2	p-value
Mean age (years)		46.8 ± 11.2	44.5 ± 15.0	0.5490
Gender (%)	male	64.0	68.0	0.7653
	female	36.0	32.0	
Histology (%)	AVM	12.0	4.0	0.2419
	Metastasis	4.0	4.0	
	WHO grade I	0.0	8.0	
	WHO grade II	12.0	32.0	
	WHO grade III	20.0	8.0	
	WHO grade IV	52.0	44.0	
Mean tumor diameter (cm)		3.5 ± 1.6	4.0 ± 1.5	0.2393
Median preoperative Karnofsky performance status (%)		90 (95% CI 87.6 – 92.4)	90 (95% CI 86.7 – 93.3)	0.3444
Preoperative language deficit (%)	none/mild	72.0	68.0	0.7576
	medium/severe	28.0	32.0	

Patient-related characteristics including mean age, gender, lesion type, mean lesion diameter, median initial Karnofsky performance status (KPS), and preoperative language function status are provided by this table for both patient groups.

### Pre- and intraoperative language mapping

#### Preoperative mapping by rTMS

In general, all enrolled patients successfully underwent language mapping by rTMS, and clear no-response errors were detectable during video analysis. Stimulation was tolerated well by each subject without causing any adverse events. Table 2 gives an overview on parameters such as RMT, stimulation intensity, and frequency. Additionally, discomfort during mapping is documented (Table 2).

**Table 2:** **Stimulation parameters of rTMS**

		GROUP 1	GROUP 2	p-value
RMT (% of stimulator output)		36.1 ± 8.8	33.3 ± 10.4	0.2976
Mapping intensity (% of RMT)		103.0 ± 9.6	103.0 ± 10.6	0.9711
Mapping frequency/number of pulses	5 Hz/5 pulses	17	9	0.0742
	7 Hz/5 pulses	4	9	
	7 Hz/7 pulses	4	7	
Pain (VAS)	convexity	2.0 ± 1.7	2.7 ± 1.9	0.3050
	temporal	4.6 ± 2.3	4.9 ± 2.2	

This table provides information about stimulation parameters including resting motor threshold (RMT, % of stimulator output), mapping intensity (% of RMT), stimulation train frequency, number of pulses in a stimulation train, and pain scores (according to the visual analogue scale, VAS).

**Figure 1:**
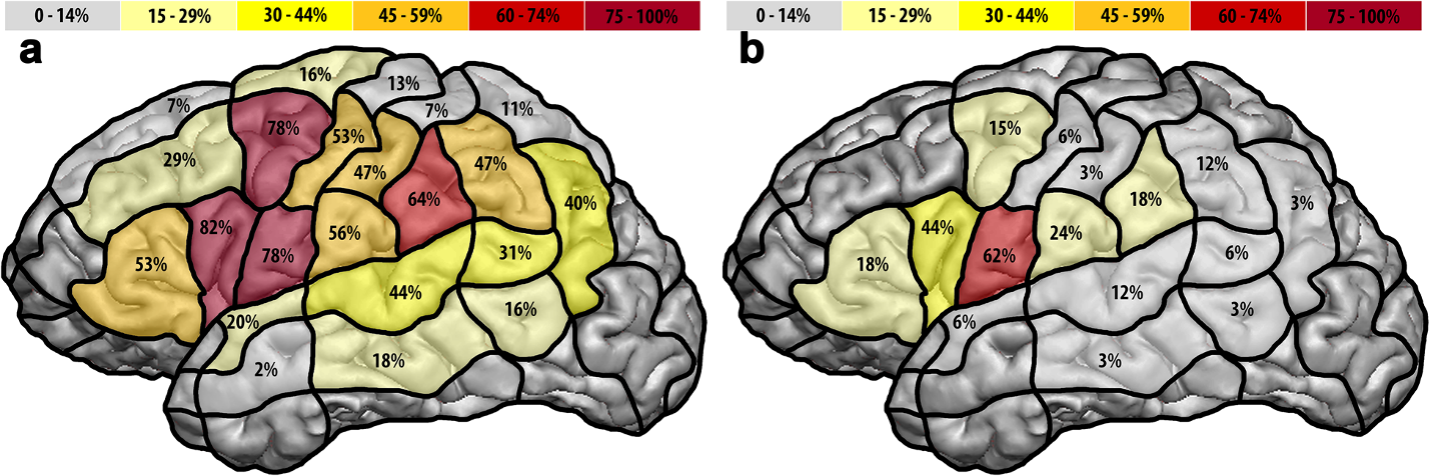
rTMS and DCS error maps. This figure graphically illustrates the language mapping results gained by preoperative rTMS **(a)** or intraoperative direct cortical stimulation (DCS) **(b)** for both patient cohorts together. The percentage results from the number of individuals with no-response errors per cortical parcellation system (CPS) region divided by the number of stimulated patients.

According to Figure 1a, which illustrates the preoperatively gained rTMS language mapping results of both patient cohorts together within the CPS, the highest rates were observed after navigated stimulation of the opercular inferior frontal gyrus (82%), ventral precentral gyrus (78%), and the posterior middle frontal gyrus (78%).

#### Intraoperative mapping by DCS

Figure 1b visualizes the intraoperatively gained DCS language mapping results of GROUP 1 and GROUP 2. The highest rates were observed after stimulation to the ventral precentral gyrus (62%) and the opercular inferior frontal gyrus (44%). Due to craniotomy limits, the spatial extent of DCS language mapping is restricted to cortical regions surrounding the brain lesion and therefore DCS cannot regularly provide the extensive language maps that rTMS provides.

### Surgery-related characteristics

#### Craniotomy size

**Figure 2:**
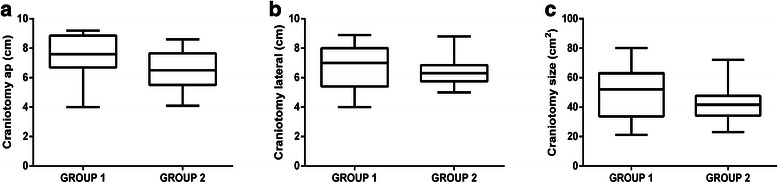
Craniotomy sizes. Boxplot of craniotomy extension for GROUP 1 compared to GROUP 2 with median, min-, and max-whiskers, and quartile-boxes for the anterior-posterior (ap) direction (**a**; p = 0.0117), lateral direction (**b**; p = 0.6430), and overall size of the craniotomy (**c**; p = 0.0373).

The ap extent was 7.5 ± 1.4 cm (median 7.6 cm, range 4.0–9.2 cm) for GROUP 1 and 6.5 ± 1.3 cm (median 6.5 cm, range 4.1–8.6 cm) for GROUP 2 patients (p = 0.0117; Figure 2a). The lateral craniotomy extent was 6.6 ± 1.5 cm (median 7.0 cm, range 4.0–8.9 cm) for GROUP 1, and 6.4 ± 1.0 cm (median 6.3 cm, range 5.0–8.8 cm) for GROUP 2 (p = 0.6430; Figure 2b). The overall craniotomy size was 50.1 ± 16.8 cm^2^ (median 52.0 cm^2^, range 21.1–80.1 cm^2^) for GROUP 1 and 41.6 ± 10.4 cm^2^ (median 41.6 cm^2^, range 23.0–72.2 cm^2^) for GROUP 2 (p = 0.0373; Figure 2c). Thus, there was a significant difference in both the ap craniotomy extent as well as the craniotomy size between GROUP 1 and GROUP 2.

#### Duration of surgery

Overall, the duration of surgery was shorter for patients in GROUP 2 in comparison to subjects in GROUP 1. Although the difference between both groups was not statistically significant, there is a clear trend towards shorter surgery duration for the patients in GROUP 2 (Table 3).

**Table 3:** **Surgery-related characteristics and postoperative course**

		GROUP 1	GROUP 2	p-value
Mean surgery duration (min)		240.3 ± 53.2	215.5 ± 48.5	0.0914
Residual tumor (%)	intraoperatively expected	12.0	24.0	0.2695
	on post-operative MRI	44.0	40.0	0.7745
	unexpected residual	32.0	16.0	0.1853
Surgery-related complications on MRI (%)	Increasing edema	4.2	0.0	0.4810
	Ischemia	29.2	30.8	
	Bleeding	25.0	15.4	
	CSF circulation dysfunction	4.2	0.0	
Median postoperative Karnofsky performance status (%)		80 (95% CI 75.8 – 84.2)	90 (95% CI 85.5 – 94.6)	0.2102
Mean inpatient stay (days)		12.2 ± 6.5	11.6 ± 5.8	0.7152
Postoperative language deficit (%)	none/mild	52.0	84.0	0.0153
	medium/severe	48.0	16.0	
Follow-up language deficit (%)	none/mild	84.0	92.0	0.3841
	medium/severe	16.0	8.0	

This table provides information about the clinical course of GROUP 1 compared to GROUP 2, including duration of surgery, residual tumor, unexpected residual, surgery-related complications, Karnofsky performance status scale (KPS), inpatient stay, language status at the 5th postoperative day, and language status during follow-ups.

#### Residual tumor

Gross total resection (GTR) according to the intraoperative judgment was achieved more often in GROUP 1 (Table 3). With regard to postoperative MRI scans, GTR was achieved at a comparable level in both groups (Table 3). However, the number of unexpected residuals was obviously higher for the patients in GROUP 1 (Table 3).

#### Peri- and postoperative complications

Regarding potential complications, increasing edema, ischemia, bleeding, and disrupted circulation of cerebrospinal fluid were taken into account. There was no significant difference in the distribution of these complications between both groups (Table 3).

### Clinical course and functional outcome

#### KPS scores

Median postoperative KPS scores were comparable between both groups without showing statistically significant differences (Table 3). Nevertheless, there was a decrease in median KPS scores when comparing the initial to the postoperative values for GROUP 1 patients, while KPS scores were more stable for GROUP 2 patients (Tables 1 and 3).

#### Inpatient stay

In total, there was no difference in mean inpatient stay between both groups (Table 3).

#### Postoperative language status

**Figure 3:**
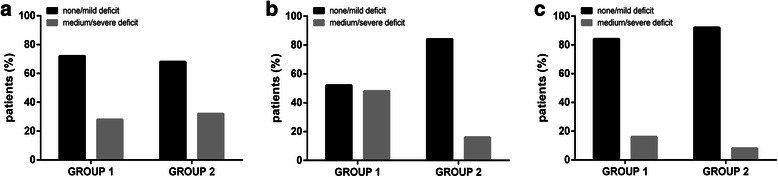
Development of language function. The graph illustrates the course of language deficits including preoperative language status (**a**; p = 0.7576), postoperative status at the 5th postoperative day (**b**; p = 0.0153), and status during follow-up 3 months after surgery (**c**; p = 0.3841) by comparing GROUP 1 with GROUP 2.

Overall, no or mild postoperative language deficits were found in 13 patients (52.0%) in GROUP 1, whereas a total number of 12 patients (48.0%) in this group showed medium to severe impairment of language at the 5th postoperative day (Table 3; Figure 3b). With regard to patients of GROUP 2, 21 subjects (84.0%) were diagnosed with no or mild postoperative deficits, and 4 subjects (16.0%) were suffering from a more severe degree of language impairment (p = 0.0153; Table 3; Figure 3b).

#### Language status during follow-up

In total, 21 patients (84.0%) in GROUP 1 presented with no or mild language impairment during follow-up 3 months after surgery, whereas 4 subjects (16.0%) of this group suffered from medium to severe language deficits (Table 3; Figure 3c). Regarding GROUP 2, 23 subjects (92.0%) showed no to mild impairment, and 2 patients (8.0%) were suffering from medium to severe deficits (p = 0.3841; Table 3; Figure 3c).

## Discussion

The investigated patient groups were highly comparable in terms of age, gender, lesion entity, lesion size, and preoperative language deficits, which should increase the comparability of the presented data in general (Table 1).

### Surgery-related characteristics

#### Craniotomy size

Preoperative rTMS language mapping seems to reduce the required size of the craniotomy, most likely due to the lack of needing to perform extensive intraoperative mapping (Figure 2a–c). Accordingly, the neurosurgeon’s intraoperative task is then primarily to confirm the preoperatively acquired rTMS data, which generally results in more circumscribed DCS-based language mapping, and therefore allows craniotomy sizes to be smaller in GROUP 2. This finding is in accordance with results published recently that showed that preoperative TMS for motor mapping in patients with motor eloquently located lesions decreases the required size of craniotomy, too [17].

#### Duration of surgery

In total, the average duration of surgery was shorter for patients in GROUP 2 when compared to their counterparts in GROUP 1 (Table 3). Data on individual language distribution was already provided by preoperative rTMS mappings and taken into account for patients in GROUP 2, and therefore the neurosurgeon was likely to be able to restrict the extent of intraoperative mapping efforts, which resulted in a shorter overall duration of surgery.

#### Residual tumor

Duffau et al., but also De Witt Hamer and colleagues reported on an increased EOR by the use of functional mapping, which proves the value of functional mapping per se—no matter whether it is performed pre- or intraoperatively [18,19]. Furthermore, two recently published studies including the impact of TMS-based motor mapping on the EOR described that GTR was more frequently achieved when preoperative TMS was performed [17,20]. However, this was not observed in the present investigation (Table 3). One possible explanation could be that the cohort size of our study is relatively small in comparison to the above-mentioned studies. Furthermore, we have to keep in mind that rTMS-based language mapping does probably not yet use its maximum potential because standardized stimulation protocols are still missing, and it is well-known that this can be regarded as a crucial point for the further development of this comparatively new field of rTMS application [4,9]. As a consequence, the basic parameters of rTMS should be examined more extensively and thoroughly in the near future, as it is likely that relatively small adjustments in the frequency or number of pulses, for example, could still improve the preoperative mapping results [4].

#### Peri- and postoperative complications

Concerning surgery-related complications, there was no significant difference between both groups (Table 3).

### Clinical course and functional outcomes

#### Development of KPS scores

There was a decrease in median KPS scores when comparing the initial with the postoperative values of GROUP 1 patients, but not for GROUP 2 patients (Tables 1 and 3). Overall, this might be primarily due to the slightly lower rate of complications found for subjects in GROUP 2 (Table 3). By now, it has already been proven that KPS scores can be considered as a prognostic indicator for survival in glioma patients [21,22]. Consequently, a positive effect of rTMS language mapping on KPS scores in general would have an obvious clinical impact.

#### Development of language function

According to the results, language deficits on the 5th postoperative day were found significantly more often for patients in GROUP 1 (Table 3; Figure 3b), although the initial, preoperative status of language function was highly comparable between both cohorts (Table 1; Figure 3a). Consequently, rTMS language mapping is likely to have played an important part in preserving language function during surgery, and EOR did not increase (Table 3).

When comparing the postoperative language impairment to the status during follow-up, most of the GROUP 1 patients improved (Table 3; Figure 3c), probably due to speech therapy. At least partly, such rehabilitation treatment might have been avoided by better immediate postoperative language outcome, which was achieved by preoperative rTMS mapping in this study.

## Conclusions

Generally, language maps can be generated by rTMS in brain tumor patients, and this approach has once again proven to be safe and well tolerated. Moreover, for the first time, the present study shows that rTMS for preoperative language mapping might even have a positive impact on the patients’ clinical course by significantly minimizing craniotomy size and reducing the rate of directly postoperative language impairment. Although these results are quite encouraging, there was only a trend observed regarding all other clinical parameters investigated in the present study. As a consequence, more studies including randomized controlled trials with larger patient cohorts are needed to further investigate the distinct impact of rTMS language mapping on the clinical course of brain tumor patients.

## Abbreviations

ap: Anterior-posterior
CI: Confidence interval
CPS: Cortical parcellation system
DCS: Direct cortical stimulation
DTI: Diffusion tensor imaging
DWI: Diffusion-weighted imaging
EOR: Extent of resection
fMRT: Functional magnetic resonance imaging
GTR: Gross total resection
KPS: Karnofsky performance status
MRI: Magnetic resonance imaging
RMT: Resting motor threshold
rTMS: Repetitive navigated transcranial magnetic stimulation
SD: Standard deviation
VAS: Visual analogue scale
